# *Aspergillus fumigatus* cholangitis in a patient with cholangiocarcinoma: case report and review of the literature

**DOI:** 10.1007/s15010-020-01487-4

**Published:** 2020-08-29

**Authors:** Kathrin Rothe, Sebastian Rasch, Nina Wantia, Alexander Poszler, Joerg Ulrich, Christoph Schlag, Wolfgang Huber, Roland M. Schmid, Dirk H. Busch, Tobias Lahmer

**Affiliations:** 1 School of Medicine, Institute for Medical Microbiology, Immunology and Hygiene, Technical University of Munich, Munich, Germany; 2 School of Medicine, Department of Medicine II, Technical University of Munich, University Hospital Rechts der Isar, Ismaninger Str. 22, 81675 Munich, Germany; 3 German Centre for Infection Research (DZIF), Partner site Munich, Munich, Germany

**Keywords:** Aspergillus cholangitis, Extra-pulmonary aspergillosis, Liposomal amphotericin B

## Abstract

*Aspergillus *spp*.* cholangitis is an uncommon presentation of invasive aspergillosis. Only few cases are described in the literature affecting severely immunocompromised patients or patients following biliary surgery. Especially, invasive aspergillosis in non-haematological patients is associated with high mortality rates, caused by atypical presentations, which is associated with a delay in diagnosis and therapy. We report a 72-year-old man with primary diagnosis of cholangiocarcinoma and stent implantation by endoscopic retrograde cholangiopancreatography (ERCP) for biliary decompression who developed severe cholangitis with invasive aspergillosis. The patient had no history of prior hospitalisation, no immunosuppressive therapy and no preceding biliary surgery. Furthermore, in this exceptional case of extrapulmonary aspergillosis, there were no signs of pulmonary involvement. From the literature review, only three cases of Aspergillus cholangitis could be identified. Clinical manifestations of invasive aspergillosis can be variable and classical risk factors such as immunosuppression are not mandatorily present. Clinical awareness of these rare cases is of vital importance for initiation of correct therapy.

## Introduction

*Aspergillus *spp*.* are ubiquitous saprophytic environmental fungi causing human disease by inhalation or ingestion of airborne conidia, which in healthy individuals are quickly removed by mucociliary clearance and alveolar macrophages [1, 2]. The most common species of *Aspergillus spp.* causing invasive disease are *Aspergillus* (*A*.) *fumigatus*, *A*. *flavus*, *A*. *niger*, *A*. *terreus*, and *A*. *nidulans* with *A*. *fumigat*us accounting for the majority of cases of invasive aspergillosis. Mortality associated with invasive aspergillosis exceeds 50% [3, 4].

Classical risk factors for invasive aspergillosis in patients include haematological malignancy, neutropenia or immunosuppressive therapy (e.g. steroids), advanced AIDS (acquired immune deficiency syndrome) or advanced neoplasia, altered lung function such as COPD (chronic obstructive pulmonary syndrome) and liver failure and liver cirrhosis [2, 5, 6]. None of these risk factors was present in this special case. Diagnosis could only be established by clinical and microbiological review of the case and by using galactomannan-antigen tests repeatedly.

In the gastrointestinal tract, especially the presentation of invasive aspergillosis as cholangitis is a rarity but has been described following biliary surgery and severe immunosuppression [7]. In our case, those clinical indicators were not present. Hence, we present an unusual case of invasive aspergillosis without classical risk factors and only non-pulmonary manifestation in the biliary tract with cholangitis.

## Case report

**Fig. 1:**
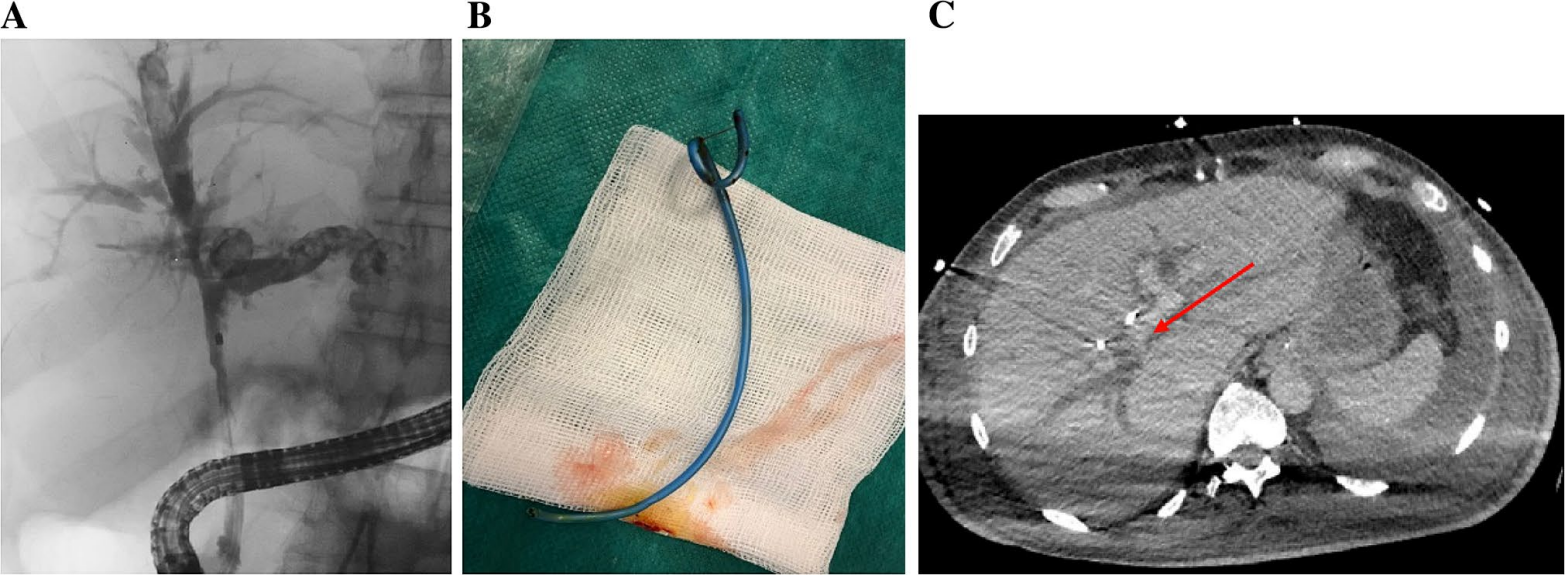
**a** ERCP of the bile duct system with cholangiocarcinoma. **b** Extracted 7 French bile duct stent. **c** CT-scan with cholangiocarcinoma (arrow)

**Fig. 2:**
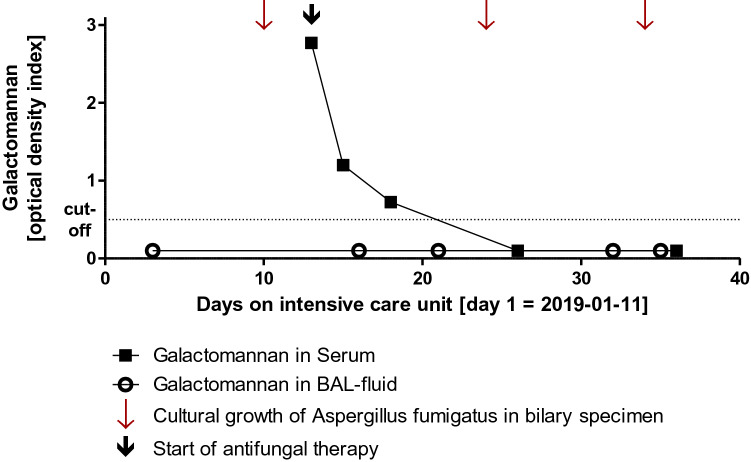
Course of results of galactomannan antigen testing

A 72-year-old Caucasian male was admitted to a hospital (collaborating institution) with newly manifested jaundice as well as a few days-history of myalgia, fatigue, recurrent fever, nausea and diarrhoea. Since the beginning of his illness, jaundice and fatigue had intensified. The patient was retired and had no relevant medical history. Furthermore, there was no history of alcohol, nicotine or drug abuse. No prior permanent medication, in particular, no antacids or proton pump inhibitors or allergies were reported. The abdominal ultrasound upon admission to the hospital revealed hepatosplenomegaly and signs of extrahepatic cholestasis with total bilirubin of 889.2 µmol/L. Therefore, in the outward hospital an endoscopic retrograde cholangiopancreatography (ERCP) with stent implantation in the bile duct became necessary because of a stenosis in the ductus hepaticus communis. One day after the procedure, the patient developed a massive post-ERCP pancreatitis with multiple organ dysfunction and was referred to our intensive care unit (ICU). On admission to our ICU, the patient was intubated and mechanically ventilated, anuric with the need for dialysis and hemodynamically instable with norepinephrine dosages of up to 2 mg/h (MAP 72 mmHg). White-cell blood count was 38.4 G/L, C-reactive protein was 349 mg/L, Procalcitonin 2.7 µg/L, total bilirubin 507.9 µmol/L, aspartate aminotransferase was 125 U/L, alanine aminotransferase 58 U/L, gamma-glutamyltransferase 343 U/L and alkaline phosphatase 281 U/L. Due to ongoing multiple organ dysfunction caused by sepsis due to post-ERCP pancreatitis and cholangitis a CT (computed tomography)-scan was performed. Beside an increasing intrahepatic cholestasis, also a cholangiocarcinoma (Klatskin carcinoma) with gallbladder infiltration was suspected, being the initial diagnosis of the malignancy. Another few days later, another ERCP was necessary due to increasing cholestatic parameters and beginning hepatic failure. Stent occlusion required extraction and new stent implantation. Additionally, bile fluid was collected for microbiological examination. Here, *Serratia marcescens*, *Enterococcus faecium, C. parapsilosis* but also *A. fumigatus* could be culturally detected. To exclude contamination and establish invasive aspergillosis, galactomannan (GM) from serum was determined. Serum GM was relevantly elevated [optical density index (ODI) 2.77] and growth of *A. fumigatus* could not only be detected by repeated bile culture but also associated to the extracted stent (Fig. 1). Repeated GM testing from BAL remained negative and a multislice chest CT scan did not show signs of pulmonary aspergillosis. Antifungal therapy with liposomal amphotericin B (3 mg/kg/day) was initiated. In patients with liver insufficiency, liposomal amphotericin B is usually the first therapeutic option [5], also the decision to use liposomal amphotericin B was based on the need to reach stable drug concentrations under ongoing continuous renal replacement therapy. Testing revealed no azole resistance. Repeated serum GM testing and follow-up bile cultures showed response to the initiated therapy (see Fig. 2). Regrettably, the patient died after 36 days on our ICU due to ongoing multiple organ dysfunction.

## Discussion

Based on the clinical and mycological findings, we report a case of invasive aspergillosis of the biliary tract caused by *A. fumigatus*.

Fungal infections of the biliary tract are uncommon. Typically, these infections are caused by *Candida *spp., which can lead to biliary obstruction and cholangitis.

Typically, invasive aspergillosis is found in immunosuppressed patients. Although, there is a growing awareness that patients without classical risk factors could be affected by invasive aspergillosis, e.g. patients with COPD or liver cirrhosis, mortality in these patients is still high because of potential atypical presentation, which is associated with delay in diagnosis and therapy.

*Aspergillus *spp*.* are ubiquitously found in the environment and acquisition and transmission is typically through the airborne route. Therefore, the lung is the most common site of invasive aspergillosis, but primary infection of non-pulmonary sites can occur in rare circumstances [3].

Based on the described case of *Aspergillus *spp*.* Cholangitis, we performed a literature review using the PubMed database to identify biliary involvement in invasive aspergillosis. Only three case reports describing aspergillosis of the biliary tract could be identified (Table 1) [8–10].

**Table 1:** Clinical course, therapy and risk factors for Aspergillus cholangitis of cases identified in the literature review

	Age, Gender	Host factors	Clinical manifestation	Microbiologic result	Treatment	Prognosis
García-Ruiz et al. [9]	Female, 34 years	Acute myeloid leukaemia Chemotherapy Neutropenia Imipenem, amikacin, teicoplanin (for 2 weeks)	Abdominal pain, jaundice, elevated liver enzymes CT-scan: multiple hepatic micronodules and cysts → PTC, biliary drainage	*A. fumigatus* in bile culture No pulmonary or disseminated aspergillosis	56-days of amphotericin B followed by Itraconazole	Negative bile cultures Clinical, radiographic improvement Death after 3 months (leukaemia relapse)
Erdman et al. [8]	Female, 19 years	No immunosuppression Compromised hepatosplenic clearance (congenital biliary atresia: status post hepato-portoenterostomy with Roux-en-Y at 6 weeks of age) Ampicillin, gentamicin, metronidazole, trimethoprim- sulfamethoxazole (for weeks)	Midgastric pain, jaundice, elevated liver enzymes CT-scan: enlarged liver, dilated intrahepatic bile ducts → PTC: placement of external biliary drain for obstruction of hepatic limb of Roux-en-Y	*A. terreus* in bile culture No pulmonary or disseminated aspergillosis	7 weeks of amphotericin B followed by itraconazole Relapse after 6 weeks: again amphotericin B	Death after 5 months (liver failure)
Yuchong et al. [10]	Male, 55 years	Status post liver transplantation Mycophenolate mofetil, prednisone, sulperazone, vancomycin, fluconazole (prophylaxis)	Fever, abdominal tenderness Emergency laparotomy: bleeding from porta hepatis, common bile duct necrosis, bile leakage	*A. flavus* in bile culture No information on dissemination	No information on therapy	Death after 2 days

*CT* computed tomography, *AML* Acute myeloid leukaemia, *PTC* percutaneous transhepatic cholangiography, *ABCD* Amphotericin B colloidal, *HBV* Hepatitis B virus infection

Involvement of the gastrointestinal tract in aspergillosis represents a possible site of infection and may occur via hematogenous dissemination from a primarily pulmonary site or via direct ingestion [11, 12]. This could be excluded by radiological diagnostic and via negative GM in bronchoalveolar lavage-fluid in our case.

One case describes aspergillosis of the liver without signs of pulmonary- or disseminated aspergillosis but presence of classical risk factors for invasive aspergillosis: Acute myeloid leukaemia with chemotherapy and neutropenia [9].

The presented patient in our case was not neutropenic but in the literature, visceral aspergillosis without clinical pulmonary involvement is conceivable rather only in severely neutropenic patients [1, 13].

The other possibility, as reported in the cases of biliary tract invasive aspergillosis, is attributable to invasive procedures at the biliary tract like surgery and external drainage (PTCD).

The reported patients in these cases underwent hepato-biliary surgery before development of Aspergillus cholangitis [8, 10]. In one case portoenterostomy explicitly served as a bidirectional conduit between intrahepatic biliary system and the small intestine allowing intestinal flora to access the intrahepatic biliary system (see Table 1).

In our case, we assume ascension of the pathogen after ERCP and stent implementation from the small intestine.

In the gastrointestinal tract, especially the small intestine is the most affected organ in reported cases of visceral aspergillosis, causing intestinal ischemia and peritonitis or liver affection through the portal venous system [1, 9, 10, 15–17].

For diagnosis and screening purposes, GM-antigen tests in serum are recommended but their sensitivity is significantly lower in non-neutropenic patients [5, 14] as neutropenia could cause an increased fungal load and facilitate angioinvasion. Also, serum neutrophils may eliminate GM [15]. Moreover, the value of GM-antigen testing has not been studied in the context of invasive aspergillosis of the digestive tract based on the rarity of this infection site [11, 16].

There is the possibility of false positive results in GM-testing in patients with piperacillin-tazobactam therapy (due to its derivation from natural compounds produced by the genus *Penicillium* [17]) and after translocation from the gastrointestinal tract after nutritional intake of GM-containing food which also includes certain enteral nutritional supplement [18]. These settings however were not present in our case.

In our case GM detection (Platelia™ Aspergilllus Ag, Bio-Rad Laboratories, Munich, Germany) was performed in BAL-fluid and in serum samples. Results were reported as ODI with a cut-off of 0.5 [5]. Moreover, primary microbiological bile cultures were performed on Columbia agar, Schaedler agar, chocolate agar (prepared culture media, Becton Dickinson, Sparks, MD, USA) and thioglycolate broth (Oxoid™ Thermo Fisher Scientific™, Waltham, MA, United States of America) as enrichment culture. In addition, culture of vortexing fluid of removed bile duct stent components was performed to increase diagnostic sensitivity and to establish association of *Aspergillus* growth with stent components. *Aspergillus* was then sub-cultured on sabouraud-dextrose-agar (Oxoid™ Thermo Fisher Scientific™, Waltham, MA, USA) for species identification: macroscopic, microscopic and via MALDI-TOF (Bruker Daltronics GmbH, Leipzig, Germany).

Yet, in our case, serial serum GM-antigen testing showed the response to antifungal therapy and helped to establish the diagnosis of extra-pulmonary aspergillosis in the first place emphasising the potential value of this diagnostic tool for extrapulmonary aspergillosis.

Treatment of extrapulmonary aspergillosis could be challenging and antifungal resistance is increasing.

Therefore, phenotypic screening for azol-resistance was performed using RPMI (Becton Dickinson, Sparks, MD, United States of America) agar plates supplemented with voriconazole and itraconazole and an antifungal-free agar as growth control. Azole susceptible isolates would have been identified by growth on the antifungal-free agar and absence of growth on plates containing azoles [19].

To date, the antifungal agents licenced for the first line treatment of invasive aspergillosis include voriconazole, isavuconazole and amphotericin B and its lipid formulations. However, as also stated in the latest ESCMID-ECMM-ERS guidelines, liposomal amphotericin B is usually the first therapeutic option in patients with liver insufficiency. In this case, we used liposomal amphotericin B because of the need for continuous renal replacement therapy and the impaired liver function to reach stable drug concentrations [16, 17]

The role of new antifungals in liver insufficiency e.g. isavuconazole has to be evaluated in future studies as promising data in patients with neutropenia or allo-HSCT are already available.

## Conclusion

Clinical manifestations of invasive aspergillosis can be variable and classical risk factors such as immunosuppression are not mandatorily present. Clinical awareness of these rare cases is of vital importance for initiation of correct therapy.
